# Association between inflammatory bowel disease and interleukins, chemokines: a two-sample bidirectional mendelian randomization study

**DOI:** 10.3389/fimmu.2023.1168188

**Published:** 2023-05-09

**Authors:** Guojiu Fang, Fanzhi Kong, Haiqing Zhang, Bin Huang, Jifa Zhang, Xueli Zhang

**Affiliations:** Department of General Surgery, Shanghai Fengxian Central Hospital, Shanghai, China

**Keywords:** inflammatory bowel disease, interleukins, chemokines, mendelian randomization, genome-wide association study (GWAS)

## Abstract

**Background:**

Mendelian randomization (MR) was used to evaluate the bidirectional causal relationship between inflammatory bowel disease (IBD) and interleukins (ILs), chemokines.

**Methods:**

Genetic instruments and summary data of five ILs and six chemokines were obtained from a genome-wide association study database, and instrumental variables related to IBD were obtained from the FinnGen Consortium. Inverse variance weighting (IVW) was used as the main MR analysis method, and several other MR methods including MR-Egger and weighted median were used to confirm the reliability of the results. Sensitivity analyses such as heterogeneity and pleiotropy were also performed.

**Results:**

The IVW method provided evidence to support that genetically predicted IL-16, IL-18, and CXCL10 significantly positively correlated with IBD, while IL-12p70 and CCL23 significantly negatively correlated with IBD. IL-16 and IL-18 had a suggestive association with an increased risk of ulcerative colitis (UC), and CXCL10 had a suggestive association with an increased risk of Crohn’s disease (CD). However, there was no evidence to support that IBD and two main subtypes (UC and CD) are associated with changes in the levels of ILs and chemokines. The results of the sensitivity analyses were robust and no evidence of heterogeneity and horizontal pleiotropy was observed.

**Conclusions:**

The present study showed that some ILs and chemokines affect IBD, but IBD and its main subtypes (UC and CD) have no effect on the level changes of ILs and chemokines.

## 1 Introduction

Inflammatory bowel disease (IBD), including Crohn’s disease (CD) and ulcerative colitis (UC), is a nonspecific immune-mediated, chronic recurrent gastrointestinal disease (1). In recent years, the incidence of IBD has maintained a growing trend in developing countries in Asia, South America, the Middle East, Africa, and other regions (2). With prevalence rates currently projected to double between 2020 and 2025, IBD is already a global disease, which will negatively affect social health care and the economy (3).

IBD is a group of heterogeneous diseases that may involve the host immune system, genetic variability, and environmental factors (4). Most studies have focused on the role of immune responses in the pathogenesis of IBD (5, 6). Dysregulation of homeostasis of various cytokines [e.g., interleukins(ILs), chemokines, tumor necrosis factors, etc.] in the body leads to recurrent intestinal inflammation (7, 8). ILs are important cytokines involved in a variety of inflammatory pathways, and there have been many studies on their roles in the development, pathogenesis, and treatment mechanism of IBD (9). For instance, a prospective observational study examined the expression of mucosal cytokines in 55 UC patients and detected the expression level of 10 types of ILs after their recurrence, and confirmed that IL-8 is the most potent predictor of UC recurrence (10). Meanwhile, clinical research evidence also supports the role of various chemokines in the pathogenesis of IBD. For example, Dr. Raja Fayad’s team detected the concentration of various chemokines in the serum of 18 CD patients, 24 chronic CD patients, and 10 healthy controls. The researchers found that a series of chemokines in IBD patients were significantly increased compared with normal healthy donors, including CCL25, CCL23, CXCL5, CXCL13, CXCL10, and CXCL11, etc (11). Although several studies have shown the relationship between ILs, chemokines and IBD, there is a lack of consensus and comprehensive evaluation of the findings. A larger sample size and reliable methods are still needed to analyze and evaluate the interaction between ILs, chemokines and IBD. Furthermore, observational studies have some potential limitations, such as residual confounding and reverse causality, and lack of high-quality data from randomized trials.

To address the the problems with observational studies, we use a Mendelian randomization (MR) method to assess the causal relationship between exposure and outcome in our study. MR regards genetic variation as an instrumental variable (IV), genetic variation is naturally and randomly assigned to offspring at conception, and it can avoid the influence of common confounding factors, such as environmental factors. With the continuous progress of scientific research and technology, genome-wide association study (GWAS) databases are increasingly enriched and shared, providing a large number of IVs available for MR analysis methods. In this study, we used MR analysis to assess the two-way causal relationship between multiple ILs (IL-8, IL-12, IL-16, IL-17, IL-18), chemokines (CCL20, CCL23, CCL25, CXCL5, CXCL10, CXCL11), and IBD and its subtypes. It is expected to provide some theoretical basis for basic research.

## 2 Methods

### 2.1 Study design

This study is based on a database of genetic associations from GWAS summary datasets (https://gwas.mrcieu.ac.uk/). GWAS data for ILs and chemokines were obtained from the European Bioinformatics Institute database, and GWAS data for IBD was obtained from FinnGen biobank analysis round 5 (**Supplementary Tables 1**). We first performed forward MR analysis to investigate the effects of six ILs and six chemokines on IBD risk. Secondly, we performed reverse MR analysis to examine whether genetic predisposition to IBD affected the levels of these ILs and chemokines.

### 2.2 Selection of genetic instruments

Single nucleotide polymorphisms (SNPs) strongly associated with exposures were selected at the genome-wide significance level of *p* < 5×10^-6^. SNPs in linkage disequilibrium were extracted for independence with a clumping algorithm in PLINKv1.9 (http://www.cog-genomics.org/plink/1.9/), and relevant parameters were set as a cutoff of R^2 = ^0.001 and kb = 10000. SNPs missing in the outcome database were replaced with SNPs with strong linkage disequilibrium (LD) (R^2^ > 0.8), and SNPs that failed to find alternative sites were excluded.

### 2.3 Statistical analysis

F statistics were used to identify weak instrument bias risks, and MR analysis was considered favorable when the F statistic was greater than 10. This study is a two-sample bidirectional mendelian randomization analysis. Main MR analysis was conducted using the inverse variance weighted (IVW) method. For exposures proxy by one or two SNPs, IVW with fixed effects was used. Multiplicative random effects were used for MR analysis based on more than three SNPs or heterogeneity. Other MR methods used to check the consistency of the results include the weighted median, MR-Egger, the simple mode, and the weighted mode. The heterogeneity of SNPs was assessed by the Cochran Q test analysis of IVW and MR-Egger. The MR-Egger intercept test was used to detect the presence of horizontal pleiotropy. Bidirectional two-sample MR analysis was performed using the TwoSampleMR (version 0.5.6) package in R software (version 4.1.2).

## 3 Results

### 3.1 Forward MR analysis

**Figure 1:**
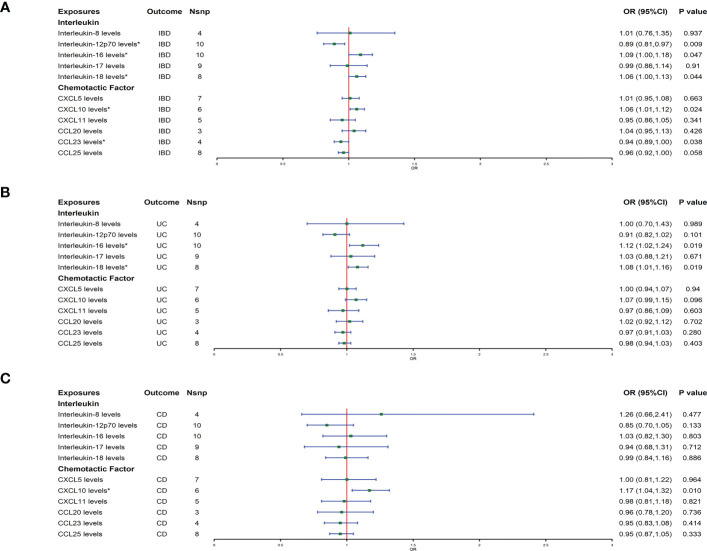
**(A**–**C)** represent mendelian randomized estimates of the causal effects of IBD and its two major subtypes (UC, CD) on ILs and chemokines, respectively. Estimates are presented as odds ratios (ORs) and 95% CIs from bidirectional mendelian randomization analyses. OR, odds ratio. 95% CI, 95% confidence interval. * indicates that there is a causal relationship between exposure and outcome.

The result of the MR analysis showed that genetically predicted IL-16 [odds ratio (OR): 1.09; 95% confidence interval(CI): 1.00–1.18; *p* = 0.047] and IL-18 (OR: 1.06; 95% CI: 1.00–1.13; *p* = 0.044) significantly positively correlated with IBD, and IL-12p70 (OR: 0.89; 95% CI: 0.81–0.97; *p* = 0.009) significantly negatively correlated with IBD (**Figure 1A**). For chemokines, genetically predicted levels of CXCL10 (OR: 1.06; 95% CI: 1.01–1.12; *p* = 0.024) were associated with the risk of IBD in the IVW model, and CCL23 (OR: 0.94; 95% CI: 0.89–1.00; *p* = 0.038) was associated with of IBD protection. The IVW results were consistent with the trend of the median weight model, although the median weight model did not reach statistical significance. Genetically predicted levels of the other ILs and chemokines were not associated with IBD risk (**Figure 1A**). In analyses of the subtypes of IBD, MR analysis showed that IL-16 (OR: 1.12; 95% CI: 1.02–1.24; *p* = 0.019) and IL-18 (OR: 1.08; 95% CI: 1.01–1.16; *p* = 0.019) had a suggestive association with increased risk of UC (**Figure 1B**), and CXCL10 (OR: 1.17; 95% CI: 1.04–1.32; *p* = 0.01) had a suggestive association with increased risk of CD (**Figure 1C**). The sensitivity analysis results showed that the MR-Egger regression analysis suggested the absence of horizontal pleiotropy, and the Cochran Q test revealed the absence of heterogeneity among IVs (**Supplementary Tables 2**, **3**).

### 3.2 Reverse MR analysis

**Figure 2:**
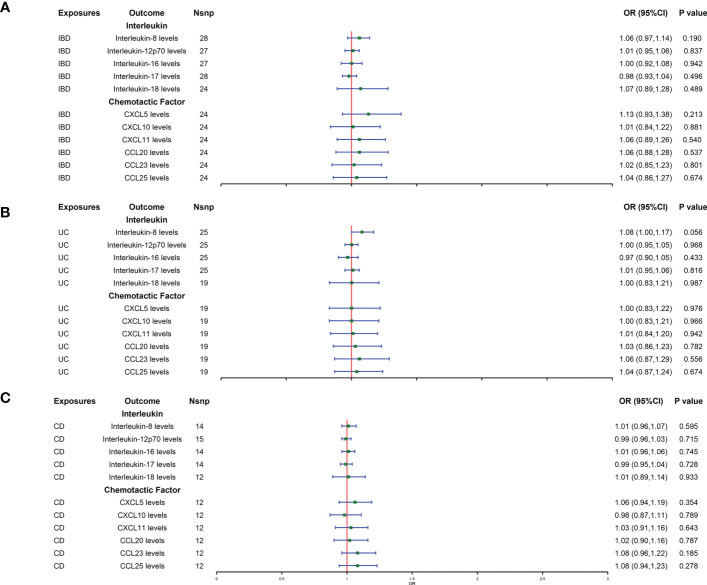
**(A**–**C)** represent mendelian randomized estimates of the causal effect of the ILs and chemokines on IBD and its two main subtypes (UC, CD), respectively. Estimates are presented as odds ratios (ORs) and 95% CIs from bidirectional mendelian randomization analyses. OR, odds ratio. 95% CI, 95% confidence interval. * indicates that there is a causal relationship between exposure and outcome.

Reverse MR analysis showed that IBD did not affect the protein levels of interleukins and chemokines (**Figure 2A**). The IBD main subtypes, UC and CD, also did not affect the levels of interleukins and chemokines (**Figures 2B, C**). Heterogeneity and horizontal pleiotropy were not detected in the sensitivity analysis (**Supplementary Tables 2**, **3**).

## 4 Discussion

To understand the causal relationship between IBD and cytokines (which includes ILs and chemokines), we used publicly aggregated GWAS data for two-way MR analysis. The forward MR results showed that the increased levels of IL-16, IL-18, and CXCL10 increase the risk of IBD, while IL-12p70 and CCL23 reduce the risk of IBD. For IBD subtypes, increased levels of IL-16 and IL-18 also increase the risk of UC, and CXCL10 increase the risk of CD. In our reverse MR analysis, there is no MR evidence indicating that IBD and its subtypes UC and CD affect the levels of these interleukins and chemokines.

Consistent with the results of our MR analyses, some meta-analyses have also demonstrated that IL-18 and IL-16 increase the risk of IBD (12, 13). Both IL-16 and IL-18 are proinflammatory cytokines, a large number of studies have shown that IL-16 and IL-18 are associated with the worsening of infectious, immune-mediated and autoimmune inflammatory diseases. Such ailments including specific dermatitis, systemic lupus erythematosus, neurodegenerative diseases and IBD (14–16). Clinical studies have shown that the increased secretion of IL-18 is related to the increased severity of IBD (17). Murine models have proved that IL-18 can destroy the mucosal barrier, trigger inflammation and magnify the damage to intestinal epithelial cells during the course of the disease (18). D Seegert et al. demonstrated that IL-16 is overexpressed in the inflamed colonic mucosa of IBD patients, and that IL-16 may contribute to the inflammatory process in IBD by promoting the recruitment and activation of inflammatory CD4+ cells and by inducing the expression of other important pro-inflammatory cytokines (19). And a study using the *Tetraodon nigroviridis* fish model proved that IL-16 can induce colitis by upregulating the expression of peptide transporter 1 (PepT1) in the colon, thereby increasing formyl-methionyl-leucyl-phenylalanine (fMLF) transport, thus triggering downstream inflammatory pathways (20).

For CXCL10, a retrospective analysis of 40 IBD patients (30 UC, 10 CD) found that patients with elevated CXCL10 levels in serum had relapses (21). In another study, colitis was induced in B6 IL-10 mice, and treatment with anti-CXCL10 during colitis development decreased clinical and histologic disease severity (22). CXCL10 induces a Th1 response in mesenteric lymph node cells and promotes effector cell recruitment in inflamed intestinal tissues (23). Zhao et al. also demonstrated that unlike conventional modulators of immune cell recruitment, the CXCL10/CXCR3 axis mediates monocyte activation and promotes tissue inflammatory responses by inducing an innate immune response (24). This all indicated that CXCL10 plays an important role in the pathogenesis of IBD.

Previous studies have demonstrated that altered levels of IL-12p70 and CCL23 in patients with IBD (11, 25). The causal relationship between IL-12p70 and CCL23 and IBD has not been reported in the relevant literature, and this study is the first to demonstrate that IL-12p70 and CCL23 reduce the risk of IBD by MR analysis.The studie has only speculated that IL-12p70 affects the body’s inflammatory response by inducing Th1 cells to differentiate and mature during IBD based on the results (25). There are no studies to explore whether and how IL-12p70 and CCL23 reduce the risk of IBD, so further basic and clinical studies are needed to confirm this.

Our reverse MR results, it supports that IBD and its subtypes do not affect the protein level of ILs and chemokines, which is inconsistent with many studies at present. For example, many studies have shown that in diseased patients, compared with healthy control individuals, the mRNA and protein levels of IL-8, IL-10, IL-12p70, IL-16, and IL-18 in pathological intestinal tissues and systemic circulation of UC and CD patients are significantly altered (20, 26–30). Controlled clinical studies have also shown that CCL20, CXCL10, CXCL16, and CCL25 are more highly expressed in inflamed intestinal tissues than in normal intestinal tissues (31), and IBD patients have a significant increase in an array of chemokines including CCL25, CCL23, CXCL5, CXCL13, CXCL10, CXCL11, and CCL21 in IBD patients as compared to normal healthy donors (11, 32). For the inconsistency between the results of different studies, we consider that ILs and chemokines play an important role in the occurrence of IBD, but the changes in their protein levels may be the result of the chain reaction of inflammation, cell damage, or microbial population changes during the occurrence of IBD, not the result of being affected by IBD.

In the process of inflammatory occurrence and development of IBD, the role of ILs and chemokines is complex and may be interactive, but MR analysis can exclude the impact of their interaction and evaluate the relationship between IBD and them only from a genetic perspective. Our research also has some limitations. First of all, this research result is the product of statistical analysis, more basic research and clinical research are needed to support our findings. Secondly, the population restrictions on individuals of European descent minimize the deviation of population structure, however, this may limit the universality of our findings in other populations.

## 5 Conclusion

The publicly available data information from the GWAS database was sourced and analyzed in this study to evaluate the causal relationship between IBD and ILs, and IBD and chemokines, by bidirectional MR analysis. Our results have shown that increased levels of IL-16, IL-18, and CXCL10 increase the risk of IBD, while IL-12p70 and CCL23 reduce the risk of IBD. However, the results of this MR study do not support that IBD can affect the level of ILs and chemokines. The potential mechanism of these results is still unclear, and further research is needed to verify our findings.

## Data availability statement

The original contributions presented in the study are included in the article/**Supplementary Material**. Further inquiries can be directed to the corresponding authors.

## Author contributions

XZ and JZ conceived the design of the study. GF and FK obtained the data and performed the data analyses. HZ and BH drafted and revised the manuscript, and all authors approved the manuscript and provided relevant suggestions. All authors contributed to the article and approved the submitted version.
